# Neonatal systemic juvenile Xanthogranuloma with Hydrops diagnosed by Purpura skin biopsy: a case report and literature review

**DOI:** 10.1186/s12887-021-02632-0

**Published:** 2021-04-06

**Authors:** Yohji Uehara, Yuka Sano Wada, Yuka Iwasaki, Kota Yoneda, Yasuhisa Ikuta, Shoichiro Amari, Hidehiko Maruyama, Keiko Tsukamoto, Tetsuya Isayama, Kenichi Sakamoto, Yoko Shioda, Osamu Miyazaki, Rie Irie, Takako Yoshioka, Naoko Mochimaru, Kazue Yoshida, Yushi Ito

**Affiliations:** 1 Department of Neonatology, Center for Maternal-Fetal, Neonatal and Reproductive Medicine, National Center for Child Health and Development, 2-10-1 Okura, Setagaya-ku, Tokyo, 157-8535 Japan; 2 Chilldren’s Cancer Center, National Center for Child Health and Development, 2-10-1 Okura, Setagaya-ku, Tokyo, 157-8535 Japan; 3 Department of Radiology, National Center for Child Health and Development, 2-10-1 Okura, Setagaya-ku, Tokyo, 157-8535 Japan; 4 Department of Clinical Pathology, National Center for Child Health and Development, 2-10-1 Okura, Setagaya-ku, Tokyo, 157-8535 Japan; 5 Department of Pathology, Nippon Koukan Hospital, 1-2-1 Koukandouri, Kawasaki-ku, Kawasaki City, Kanagawa 210-0852 Japan; 6 Department of Dermatology, National Center for Child Health and Development, 2-10-1 Okura, Setagaya-ku, Tokyo, 157-8535 Japan

**Keywords:** Systemic juvenile xanthogranuloma, Purpura, Skin biopsy, Neonate, Fetal hydrops

## Abstract

**Background:**

Systemic juvenile xanthogranuloma is a very rare disease typically presents as skin lesions with yellow papules or nodules and is sometimes fatal. We report a case of congenital neonatal systemic juvenile xanthogranuloma with atypical skin appearance that made the diagnosis difficult.

**Case presentation:**

A preterm Japanese female neonate with prenatally diagnosed fetal hydrops in-utero was born with purpuric lesions involving the trunk and face. Since birth, she had hypoxemic respiratory failure, splenomegaly, anemia, thrombocytopenia, coagulopathy, and was transfusion dependent for red blood cells, fresh frozen plasma, and platelets. Multiple cystic lesions in her liver, part of them with vascular, were detected by ultrasound. A liver biopsy was inconclusive. A skin lesion on her face similar to purpura gradually changed to a firm and solid enlarged non-yellow nodule. Technically, the typical finding on skin biopsy would have been histiocytic infiltration (without Touton Giant cells) and immunohistochemistry results which then would be consistent with a diagnosis of systemic juvenile xanthogranuloma, and chemotherapy improved her general condition.

**Conclusions:**

This case report shows that skin biopsies are necessary to detect neonatal systemic juvenile xanthogranuloma when there are organ symptoms and skin eruption, even if the skin lesion does not have a typical appearance of yellow papules or nodules.

## Background

Juvenile xanthogranuloma (JXG) is a rare benign histiocytic disorder with solitary or multiple skin lesions that present as yellow papules or nodules, which typically appear in the first year of life and are self-limiting [1–4]. JXG can be easily suspected from the yellow papules or nodules, and diagnosis is confirmed by biopsy. Systemic JXG is a very rare disease defined as the involvement of one or more visceral organs and is sometimes fatal [2, 4]. Its symptoms depend on the affected organ and include pancytopenia, anemia, thrombocytopenia, coagulopathy, cyanosis, cholestatic liver failure, hepatosplenomegaly, and seizure [1, 4–6]. The mortality rate for JXG is especially increased in cases of liver and/or central nervous system infiltration [2, 5–7]. Early diagnosis and treatment are therefore important. Systemic JXG is also easily suspected from the yellow papule or nodule appearance. We report a case of congenital neonatal systemic JXG that was difficult to diagnose due to the atypical appearance of a purple skin rash.

## Case presentation

**Fig. 1:**
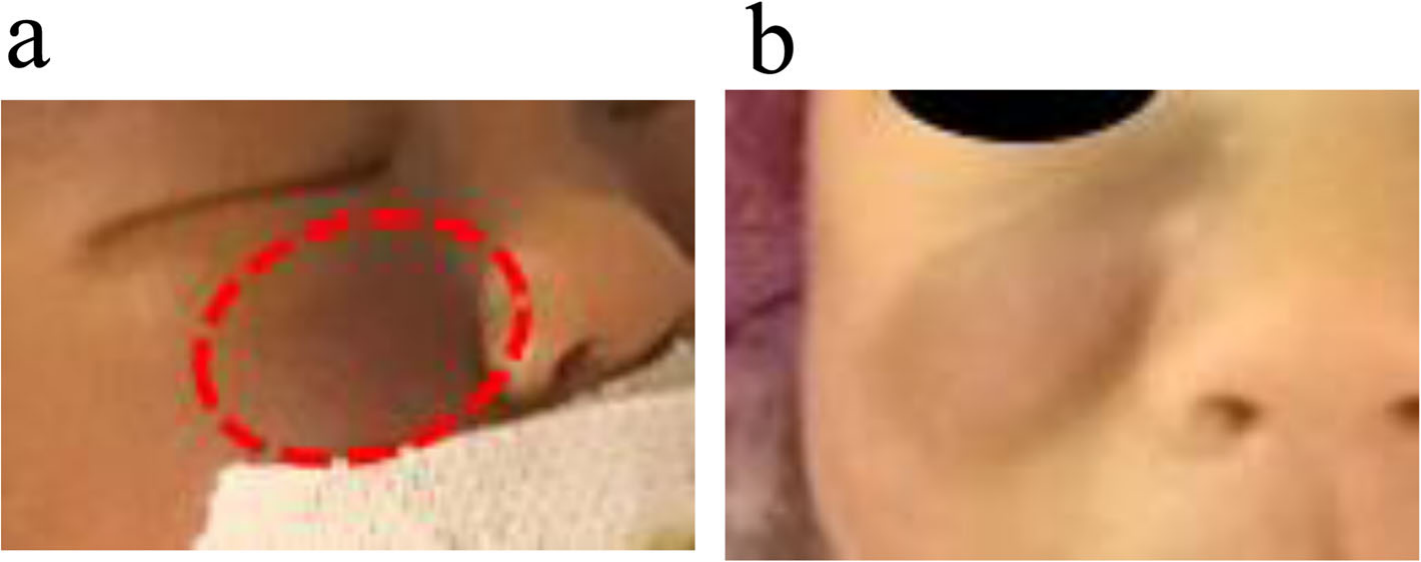
a Purpura on her face at birth. b Purpura on her face at 56 days of life. The purpura changed to a firm and solid enlarged (but not yellow) nodule

A Japanese female neonate was born by emergency cesarean section at 34 weeks of gestational age despite an uneventful prenatal course. Prenatal ultrasound (US) detected fetal hydrops and a higher middle cerebral artery peak systolic velocity (85 cm/s; 1.7 times that of the median, which was characteristic of severe fetal anemia) just before birth. The patient had a birth weight of 2362 g (+ 0.6 SD) and required mechanical ventilation immediately after birth due to hypoxemic respiratory failure. She presented severe anasarca edema, which was prominent on her trunk and face, pleural effusion and purpura on her body that included her face (Fig. 1a). She required mechanical ventilation for 52 days. Inotropes and parenteral nutrition were administered for 3 and 34 days, respectively. US showed ascites and splenomegaly but no other abnormal findings. An initial complete blood count indicated a hemoglobin level of 5.4 g/dL and a platelet count of 6 × 10^3^/μL. The patient was coagulopathic and required daily blood transfusions (antithrombin, 13.4%; D-dimer, 9.5 μg/mL; fibrin/fibrinogen degradation products, 16.6 μg/mL; the prothrombin time-international normalized ratio, activated partial thromboplastin time, and fibrinogen level were severely prolonged beyond the measurement range). With a suspicion of a hemolytic anemia or congenital infection, a Coombs test, blood, urine, and cerebral spinal fluid cultures and serologic testing for toxoplasmosis, hepatitis B, hepatitis C, syphilis, parvovirus B-19, rubella, cytomegalovirus, and herpes simplex virus, as well as polymerase chain reaction tests for varicella-zoster virus, Epstein-Barr virus, human herpesvirus 6/7, enterovirus, parechovirus, and adenovirus, were performed and results were negative. In addition, bone marrow aspiration was performed. But no leukemic blast and foamy macrophage were not seen. Thus, bone marrow aspiration also did not reveal an etiology.

**Fig. 2:**
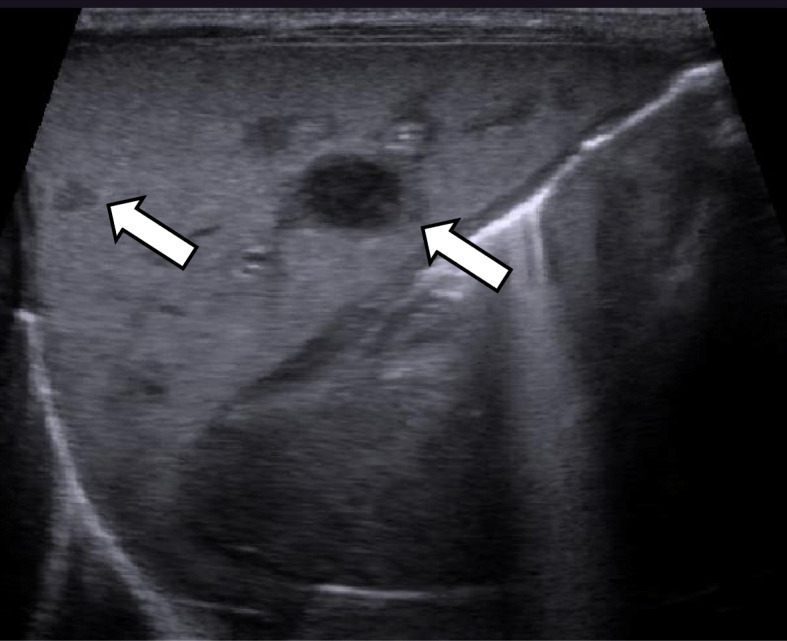
US at 10 days of life. It revealed multiple hypoechoic liver nodules (arrow)

The patient developed cholestasis and liver dysfunction with total bilirubin peaking at 30.5 mg/dL with a direct bilirubin of 21.7 mg/dL, aspartate aminotransferase of 400 IU/L, and alanine aminotransferase of 208 IU/L. At 10 days of life, an US revealed multiple hypoechoic liver nodules and ascites along with splenomegaly but no hepatomegaly (Fig. 2). At 23 days of life, magnetic resonance imaging (MRI) assessment of these liver lesions demonstrated high T2 signal intensity and high diffusion-weighted images signal and low apparent diffusion coefficient signal, and these changes were non-specific. Follow up US showed an increasing number of hypoechoic lesions with subsequent development of hepatosplenomegaly. Cardiac US, contrast-enhanced chest computed tomography, and brain MRI revealed no lesions in heart, lung and central nervous system.

**Fig. 3:**
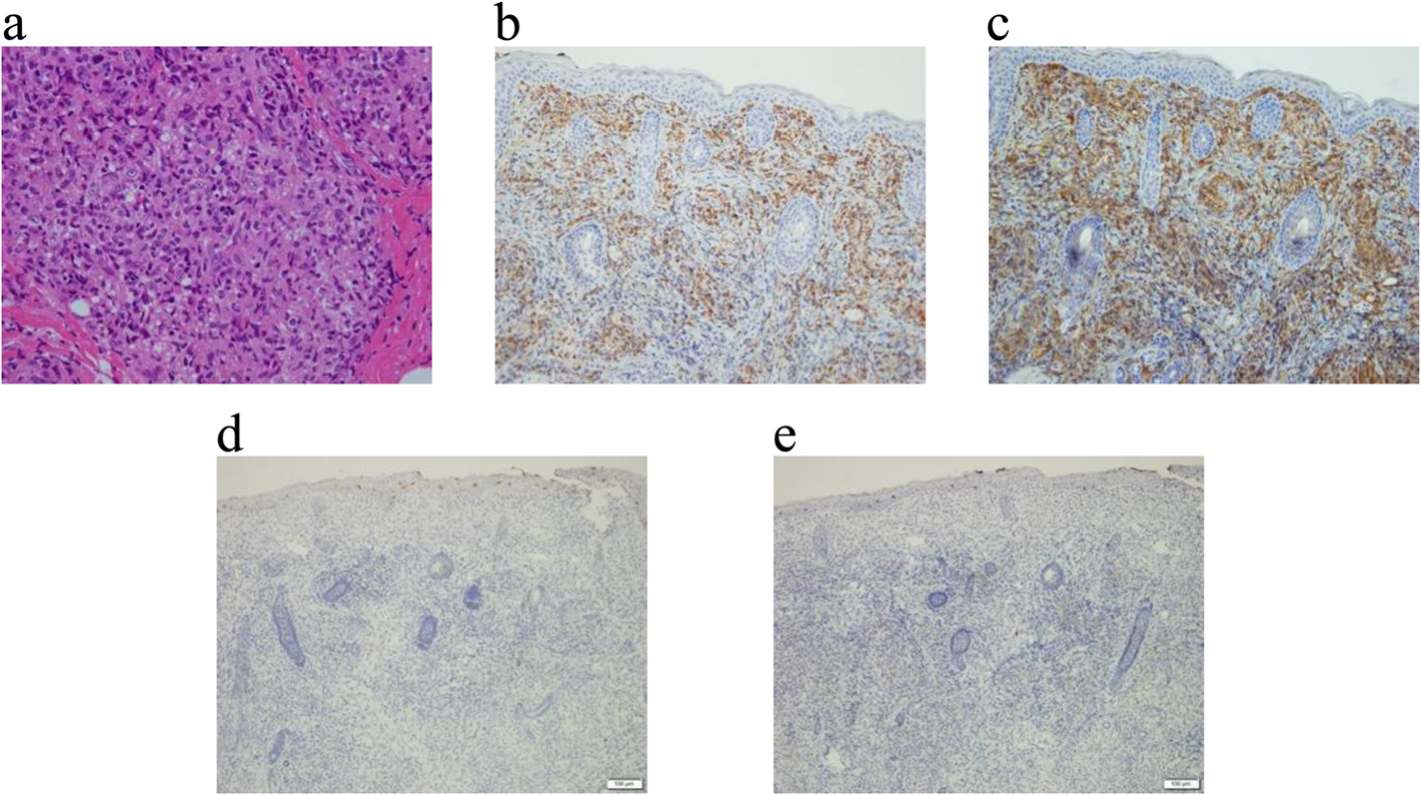
Immunohistochemistry of the skin biopsy. It revealed the expression of CD68 and CD163 in the histiocytic population, whereas there was no expression of CD1a and Langerin. Hematoxylin and eosin staining showed an accumulation of histiocytes with several foamy cells (× 200 magnification) (**a**). The immunohistochemical staining pattern of histiocytes was follows: CD68 positive (brown staining, × 100 magnification) (**b**), CD163 positive (brown staining, × 100 magnification) (**c**), CD1a negative (no brown staining, × 100 magnification) (**d**), Langerin negative (no brown staining, × 100 magnification) (**e**)

An open liver biopsy conducted at 42 days of life showed giant hepatic cell transformation, cholestasis in hepatocytes, and foamy histiocytes accumulation with no infiltration into the portal region, which did not suggest typical systemic JXG, Langerhans cell histiocytosis, hepatoblastoma, or neuroblastoma. Due to a perceived change in one of the lesions from purpuric to a more firm and enlarged nodule (Fig. 1b), a skin biopsy was performed at 56 days of life. Immunohistochemistry demonstrated that the histiocytic population was CD68+, CD163+, CD1a-, and Langerin- (Fig. 3).

Chemotherapy based on the protocol for Langerhans cell histiocytosis in Japan [8] was started at 65 days of life. Induction therapy included cytarabine, vincristine, and prednisolone. The patient received prednisolone and low-dose cytarabine; however, vincristine was withheld due to hepatic dysfunction. The multiple hepatic nodules resolved on disappeared within four months after the chemotherapy. The chemotherapy was continued for 54 weeks. The patient’s neurodevelopment and weight gain at 24 months of life was satisfactory.

## Discussion and conclusions

Making an immediate diagnosis of JXG was difficult because of the atypical appearance of the purple skin rash. At first, her purpuric skin lesions made believe that these were caused by coagulopathy. However, the lesion’s color gradually turned dark blue-purple, almost black, and the shape changed to a firm and solid enlarged nodule, these findings led to the suspicion of a tumor. A skin biopsy was performed, and the patient’s disease was finally diagnosed as JXG.

Neonatal systemic JXG is a rare disease, with only reported 32 cases in the literature (Table 1) [1, 2, 4–7, 9–32]. All cases listed in Table presented systemic symptoms or had at least one affected visceral organ within the neonatal period. JXG typically presents yellow to pink-brown papules and nodules; 16 of the 32 cases presented a typical color (such as yellow and erythematous) [1, 5, 10–12, 14–22], with three of the 16 cases subsequently changing to a typical color [5, 12, 22]. Eight cases had atypical skin lesions; one case had purpura (as in our case) [23], and the others had bluish nodules, ecchymoses, petechiae, blueberry muffin rash, and a mass [4, 6, 13, 23–27]. Six cases had unclear in the details [2, 7, 28–30, 32]. There were nine cases [5, 11, 12, 20–22, 25, 27, 28] in which the color and/or shape changed (excluding flattening, regression and disappearance) or new skin findings appeared over time, although there is a possibility that the change was not recognized because the patient was treated before the diagnosis. Change over time has been reported as a feature of congenital JXG [3]. Therefore, diagnosis by visual inspection is difficult because it involves various phenotypes such as color, shape, and changes over time. It is unclear why the emergence of congenital JXG differs from that of a typical JXG, but it has been speculated that neonatal skin has less subcutaneous fat and appears purple due to subcutaneous bleeding.

**Table 1:**
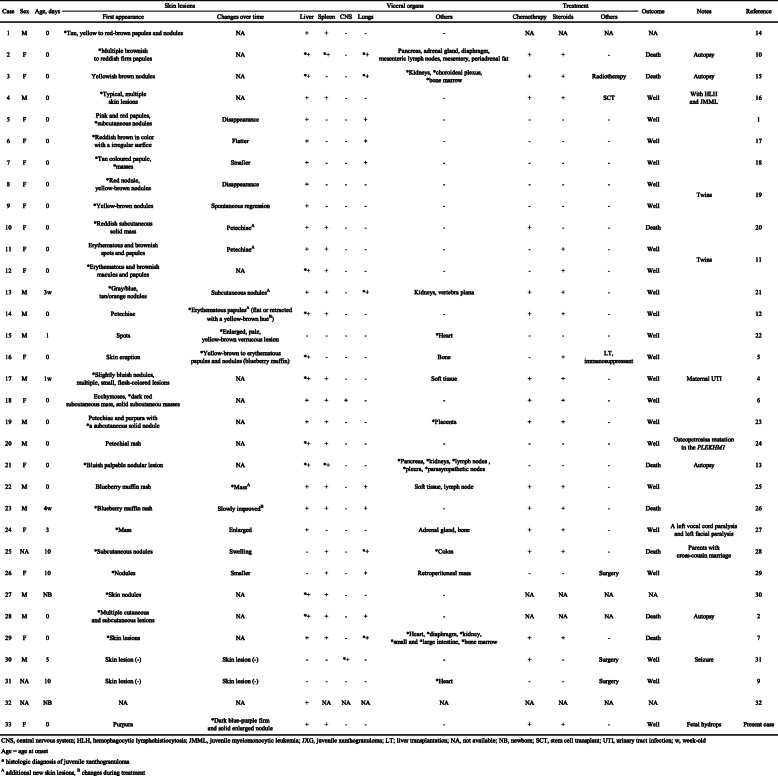
Cases of neonatal systemic juvenile xanthogranuloma. We identified 33 cases of neonatal systemic juvenile xanthogranuloma, which included one or more affected visceral organs within the neonatal period, with the detailed case information

The liver biopsy could not clearly diagnose JXG. However, we considered the results consistent with a JXG lesion because the US and MRI findings showed that it had decreased after the chemotherapy. The diagnosis of JXG could not be made in four cases through a liver biopsy [19, 20, 26], showing that liver biopsy can cause difficulties in revealing JXG and has a higher risk than skin biopsy. Congenital systemic JXG is therefore difficult to diagnose from the skin appearance.

The number of cells, including foamy histiocytes, in bone marrow aspiration after birth was too less to reveal an etiology.

When there are organ symptoms, abnormal liver findings, hydrops, and skin rash (even if the skin’s appearance is not that of a typical yellow papule or nodule), congenital JXG should be actively suspected, and a skin biopsy should be performed.

## Data Availability

The datasets supporting this article can be obtained upon request.

## Abbreviations

CNS: Central nervous system
HLH: Hemophagocytic lymphohistiocytosis
JMML: Juvenile myelomonocytic leukemia
JXG: Juvenile xanthogranuloma
LT: Liver transplantation
MRI: Magnetic resonance imaging
NA: Not available
NB: Newborn
SCT: Stem cell transplant
US: Ultrasound
UTI: Urinary tract infection
w: Week-old
